# Rush Medical College

**Published:** 1859-12

**Authors:** 


### RUSH MEDICAL COLLEGE.

It may be gratifying to the alumni and friends of this College to learn that the recent separation of several members of the Faculty has not in the slightest degree diminished its prosperity or changed the regularity of its operations. On the contrary, the present class is the largest which has attended its courses since its organization, with the exception of those of the years 1847, '48, '55 and '56. The newly elected professors are giving courses of lectures at once solid and brilliant, which recall those of the earlier years of its existence. Additions are being made to its collections. Unity of purpose to place the College in the first rank in regard to the value of its courses and the extent of its facilities prevails among all its members.

